# Nutrition Research in Aging Dogs and Cats: What We Know and What We Need to Do

**DOI:** 10.3390/ani16040571

**Published:** 2026-02-12

**Authors:** Xuan Cai, Hui Mao, Lihui Zhu

**Affiliations:** Institute of Animal Husbandry and Veterinary Science, Shanghai Academy of Agricultural Sciences, Shanghai 201106, China; caixuan1985911@163.com (X.C.); m18818111806@163.com (H.M.)

**Keywords:** aging animal, companion animal, pet nutrition, challenge, nutraceuticals, multi-modal monitoring

## Abstract

The population of dogs and cats is rapidly increasing, and their lifespans are extending due to advances in veterinary medicine and increased owner awareness. As a result, companion animals are also gradually entering an aging society. Research on the nutritional needs of aging dogs and cats remains limited. Key factors contributing to this gap include unclear biomarkers of aging, fragmented research efforts, and a lack of experimental models using healthy aged animals. By reviewing recent publicly available literature on aging biomarkers in dogs and cats, this study identifies biomarkers with practical value for aging research. It further analyzes reported physiological changes, nutritional requirements, and common dietary supplements for senior pets, and summarizes large-scale research programs focused on aging dogs and cats. Capitalizing on rapid advances in artificial intelligence, this paper proposes three key recommendations: developing aging biomarkers based on non-invasive sampling methods; accelerating the establishment of nutritional guidelines for healthy aging dogs and cats; and promoting personalized nutrition research for aging pets through cross-border and cross-regional collaboration. This study aims to consolidate existing knowledge on nutrition for aging dogs and cats, identify current research gaps, suggest directions for future studies, and provide evidence-based recommendations to support healthy aging in dogs and cats.

## Simple Summary

Advances in veterinary medicine and pet nutrition have led to an increasing aging population in dogs and cats. However, researches on the nutritional requirements of aging dogs and cats remain scarce. This review focuses on the development predicaments in research related to aging companion animals and explores the core challenges in the aging research of dogs and cats. This study examines the underlying causes of this research gap, attributing it to the ambiguity of aging biomarkers, fragmentation of relevant studies, and a lack of experimental aged animal models. In response to these three issues, we propose three strategies: developing aging biomarkers based on non-invasive sampling methods, formulating nutritional guidelines for senior dogs and cats, and establishing extensive international collaborations. This study aims to reveal the predicaments in nutritional research on senior pets, call for targeted measures to advance the development of research in this field, and ultimately facilitate the healthy aging of dogs and cats.

## 1. Introduction

A rapid expansion in the companion animal population [1] has increased the number of aging pets, particularly dogs and cats. Current estimates indicate that over one-third of the global dog and cat population are classified as senior [2]. This demographic shift is largely driven by advances in veterinary medicine, improved animal care practices, and greater owner engagement in preventive healthcare—all contributing to longer lifespans than in previous decades. Notably, record lifespans of over 31 years for dogs and 38 years cats have been reported in the past 20 years [3]. It is now common for companion pets to live into their second decade, with many surpassing 20 years of age. However, extended longevity introduces distinct health challenges, as aging is associated with a progressive decline in physiological resilience, increased susceptibility to chronic diseases, and evolving nutritional demands [4]. The aging process in companion dogs and cats parallels that in humans, characterized by sarcopenia, reduced metabolic efficiency, diminished immune function, and elevated oxidative stress [5]. Consequently, geriatric pets are frequently diagnosed with age-related conditions such as chronic kidney disease, osteoarthritis, cognitive dysfunction syndrome, and periodontal disease, all of which significantly influence their nutritional status and dietary requirements [6].

Despite these challenges, nutritional research on senior and geriatric companion dogs and cats remains limited compared to studies focused on younger adults. Many commercial pet foods persist in employing generic formulations rather than age-specific, evidence-based nutritional strategies—a practice largely attributable to the absence of well-defined nutritional standards for older pets. Notably, guidelines issued by authoritative bodies such as the Association of American Feed Control Officials, the European Pet Food Industry Federation, and the National Research Council do not differentiate between the nutritional requirements of young/adult animals and those of senior pets [3].

We identify three key factors contributing to this research gap. First, the lack of clear and reliable aging biomarkers in dogs and cats hinders accurate life-stage assessment. Second, much of the existing nutrition research is industry-driven, often characterized by limited public transparency, unclear public welfare benefits, and fragmented progress, which collectively slow the development of consensus guidelines. Third, there is a scarcity of healthy aged animals available for study, as older pets frequently present with comorbidities and are often excluded from nutritional trials in favor of clinical medical research, or studies are terminated prematurely due to insufficient subject numbers.

Though numerous reviews have addressed disease and nutrition in older animals, few have systematically examined the barriers to research and development specifically in aging companion animals. This review focuses on the development predicaments in research related to aging companion animals and explores the core challenges in the aging research of dogs and cats. Integrating the existing research findings on elderly dogs and cats, it analyzes new ideas for advancing nutritional research on aging dogs and cats in the new era. It aims to identify practical and feasible strategies for promoting such nutritional research that align with the current research context, improve the health status of elderly dogs and cats, and facilitate the harmonious development of humans and their companion animals.

## 2. Methodology for Literature Search

This study systematically reviews the existing literature on the physiological characteristics, nutritional requirements, research methodologies, and the development of anti-aging pet foods for senior dogs and cats. A comprehensive literature search was conducted primarily using the Scopus and Web of Science databases. The literature search covered the period from 1 January 1970 to 20 August 2025. Supplementary searches were performed in the China National Knowledge Infrastructure (CNKI) using corresponding Chinese keywords, such as “dog OR dogs OR canine OR canines OR cat OR cats OR feline OR felines OR pet OR pets OR “companion animal” OR “companion animals” combined with terms including “aging,” “old,” and “elder.” To specifically identify literature on aging biomarkers, the SCOPUS database was queried using the following search string: TITLE-ABS-KEY (“biomarker” OR “biomarkers” OR “biological marker” OR “biological markers”) AND (“aging” OR “ageing” OR “senescence” OR “senescent” OR “old age”) AND (“canine” OR “dog” OR “dogs” OR “feline” OR “cat” OR “cats”). The Bing search engine was also utilized for retrieving relevant web-based information.

Regarding data collection and credibility assessment, priority was given to literature indexed in the Science Citation Index (SCI), officially published books, and official documents released by authoritative bodies such as the American Association of Feed Control Officials (AAFCO) and the European Pet Food Industry Federation (FEDIAF). Chinese-language literature was considered subsequently, followed by blog articles from reputable dog and cat associations.

## 3. What Is “Aging” in Companion Dogs and Cats

### 3.1. Threshold Age for Senior Dogs and Cats

Similar to humans, a single, universally recognized definition of aging in canines and felines does not exist. Different academic institutions, organizations, and governmental entities have developed their own definitions grounded in their specific data and perspectives, but a general understanding is apparent. Table 1 provides a summary of the definitions for ‘senior dogs and cats’ as delineated by prominent international pet organizations and institutions. Senior cats are generally classified as those aged 11 years and older, whereas small, medium, and large dogs are categorized as senior at ages 11, 10, and 7 years and older, respectively. One study suggested that an 11-year-old cat is approximately equivalent to a 60-year-old human, an 11-year-old small dog corresponds to a 62-year-old human, a 7-year-old medium dog is akin to a 59-year-old human, and a 7-year-old large dog is also comparable to a 59-year-old human [7]. This is consistent with the widely held view that humans enter old age at ~60 years [8]. In practice, due to the limited availability of genuinely ‘senior’ dogs and cats for research, many scholars classify all dogs and cats aged 7 years and older as senior [9,10].

### 3.2. Biomarkers for Nutritional Status in Aging Dogs and Cats

Aging is distinct from disease; it is a gradual process that begins at birth and continues throughout life, characterized by a decline in physiological functions and adaptability [8,16]. Biomarkers play a crucial role in the nutritional assessment of elderly dogs and cats. In 2023, López-Otín et al. proposed 12 aging biomarkers, including genomic instability, telomere attrition, epigenetic alterations, loss of proteostasis, disabled macroautophagy, deregulated nutrient-sensing, mitochondrial dysfunction, cellular senescence, stem cell exhaustion, altered intercellular communication, chronic inflammation, and dysbiosis [17]. However, certain biomarkers are not applicable to dogs and cats, and aging biomarkers vary among different dog body figures [18]. As summarized in Table 2, we excluded controversial biomarkers (with inconsistent results in multiple studies) and those only studied at the omics level, selecting only biomarkers supported by at least two independent studies (except for organ-level biomarkers).

As shown in Table 2, aging biomarkers in dogs and cats can be broadly categorized into four levels, i.e., molecular, cellular, organ, and gut microbiota.

At the molecular level, the highest number of biomarkers can be observed, primarily including macromolecular damage indicators such as DNA damage, telomere length, malondialdehyde, glutathione, and 8-hydroxy-2-deoxyguanosine. As previously mentioned, these markers are largely derived from the free radical damage theory [56]. One of the main approaches in current aging research involves using antioxidant-related indicators as biomarkers [9,56,57], with typical examples including antioxidant enzyme activity and macromolecular oxidation products [56]. However, these metrics rely heavily on the free radical theory of aging, which has faced considerable scrutiny in recent years [58,59]. Moreover, the specificity of these oxidative stress biomarkers is limited, as many physiological processes, such as stress responses, can also lead to free radical imbalance.

Another category of biomarkers involves substances such as amyloid-beta, neurofilament light chain, osteoprotegerin, angiotensin II, endothelin-1, osteocalcin, and the carboxyterminal propeptide of type I procollagen, which are used to characterize age-related diseases or physiological features, including cognitive dysfunction syndrome [27], neuroacoustic injury and degeneration [29], and bone turnover [39]. Declining renal function is a significant indicator of aging in both dogs and cats [51,60]. In elderly cats, serum symmetric dimethylarginine (SDMA) and creatinine are key biomarkers for assessing renal function, which is closely linked to nutritional status. A six-month dietary intervention study involving 32 healthy geriatric cats showed that serum SDMA concentration correlates more strongly with glomerular filtration rate than serum creatinine (sCr) concentration [61]. In older cats with reduced lean body mass, SDMA levels rise with age-related declines in glomerular filtration rate, whereas sCr levels decrease due to muscle loss. Another study of 80 client-owned geriatric cats found that cats fed a test diet enriched with functional lipids, antioxidants, and other supplements maintained stable SDMA and creatinine levels, indicating preserved renal function [62]. In contrast, cats fed owner-selected diets exhibited progressive renal impairment.

These biomarkers primarily focus on specific geriatric diseases, making it difficult to distinguish between animals undergoing ‘healthy aging’ [8] and those suffering from age-related pathologies. Additionally, certain biomarkers related to cellular processes, such as cell cycle regulators (P16, P21) and epigenetic features, have been extensively studied in human medicine but remain underexplored in dogs and cats. Notably, some epigenetic-based identification methods have yet to achieve standardized application.

In recent years, omics-based approaches for screening aging markers have gained increasing attention. For example, Li et al. [31] identified 17 transcriptional and protein markers related to stem cell aging, along with five metabolic markers. Among these, Penitrem A and UDP-N-acetylglucosamine have been confirmed as reliable metabolic indicators of both individual and cellular aging, offering new perspectives for selecting aging biomarkers in dogs and cats. However, many similar omics studies lack further validation, and their reliability remains to be confirmed.

Most research at the cellular level has focus on lymphocytes. For example, Li et al. [31] employed a multi-omics approach to identify nine aging-associated cell populations and nine CD8^+^ T cell aging markers that are conserved across species. However, like other biomarkers, these cellular indicators are not exclusive to healthy aging, thereby limiting their diagnostic specificity.

Although aging affects multiple systems and organs, including the brain and joints, comprehensive organ-level studies in companion animals remain scarce. This is reflected in Table 2, which cites only one reference under organ-level biomarkers. Furthermore, most current organ-level aging assessments rely on qualitative observations [51,52,53], posing challenges for establishing quantifiable metrics. Relying solely on such markers therefore weakens the robustness of aging research.

Changes in gut function also represent an important aspect of aging. In pet nutrition research, fecal quality and gut microbiota composition are often used as key indicators of intestinal health [56,63,64]. However, gut microbiota is highly susceptible to dietary variations [63,65], which restricts its utility as a specific biomarker of aging.

The American Federation for Aging Research has proposed criteria for ideal aging biomarkers, stating that they should: (1) predict aging rate by correlating with age; (2) monitor processes underlying healthy aging; (3) be measurable repeatedly without harm; and (4) be applicable across species, including humans and animals [66]. To date, however, none of the biomarkers listed in Table 2 fully satisfies all these criteria. Current biomarkers generally lack specificity, making it difficult to differentiate between changes due to “healthy aging” and those caused by disease. Many are also influenced by factors such as diet and breed. Some even exhibit breed-specific relevance; for instance, Ekici et al. found age-related effects on serum bone alkaline phosphatase, osteocalcin, and C-terminal telopeptide of type I collagen in Kangal shepherd dogs—a working breed whose bone metabolism patterns may not reflect those of typical, less active pet dogs and cats [39].

Another issue is the pronounced imbalance in aging biomarker research between dogs and cats. While numerous studies exist on canine aging biomarkers—partly due to dogs serving as models for human aging—research on feline aging remains extremely limited. Given the distinct physiological differences between the two species [67], findings from dog studies cannot be directly extrapolated to cats.

**Figure 1:**
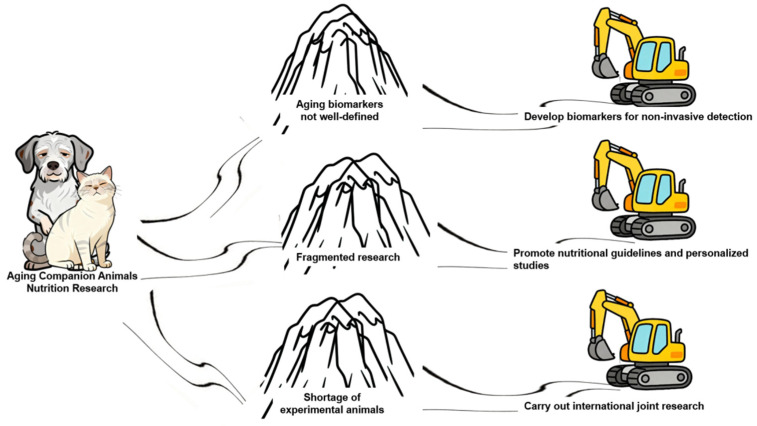
Research on nutrition in aging dogs and cats. This image illustrates the three major obstacles currently hindering research on aging companion animals, alongside feasible solutions.

In light of the ongoing ambiguity even in human aging biomarkers, a multi-method, integrated assessment approach appears to be a more prudent strategy [17]. Thus, the ambiguity of aging biomarkers constitutes the primary barrier to nutritional research on senior dogs and cats (Figure 1). Moving forward, it will be essential to refine the use of biomarkers to accurately evaluate the nutritional status of aging companion animals and to provide precise guidance for nutritional interventions.

## 4. What We Have Known About Aging Dogs and Cats

### 4.1. Physiological Characteristics of Aging Dogs and Cats

Aged companion animals undergo significant physiological changes that directly impact their nutritional needs (Table 3). Similar to humans, aging increases their susceptibility to tumors and chronic pain [68,69]. The incidence of mammary tumors, alimentary neoplasia, and brain neoplasms rises in older dogs and cats [70,71,72,73]. On average, tumors develop around 9.5 years of age (approximately 9.3 years in dogs and 10.5 years in cats), with a peak occurrence at 10 years and an overall prevalence of 14.9% in dogs and 13.2% in cats, posing a substantial threat to their wellbeing [74].

Energy requirements in older pets are an active area of research. Generally, reduced physical activity and a lower basal metabolic rate decrease their energy needs [84]. Although older animals are often thought to have diminished digestive capacity, a study on dogs aged 2.6 to 14.2 years found that age did not significantly affect fat digestibility [85]. Dogs between 6 and 12 years showed improved digestibility of crude protein, total dietary fiber, calcium, and phosphorus when fed a diet containing 6% total dietary fiber [85], indicating that nutrient absorption can be enhanced with optimally balanced fiber.

Furthermore, research indicates that senior dogs and cats enter a pathological state that may increase energy demand [15]. In elderly pets, age-related tooth loss, oral diseases, and declines in taste and smell commonly reduce appetite and intake, leading to a negative energy balance that compromises quality of life [15,75]. While digestive ability is often presumed to decline with age [69,86], several studies report that protein and fat digestibility remains unchanged or may even improve in healthy seniors [85,87,88].

Aging also alters gut microbiota structure, which can affect overall health of pets [3,89,90]. Changes in intestinal epithelial permeability in older in older cats and dogs require further investigation, as they may influence digestion, absorption, and immune function. In the musculoskeletal system, osteoarthritis and joint degeneration are prevalent, significantly impairing mobility. The nervous system in aging dogs and cats undergo considerable change, with declining cognitive function, brain atrophy, reduced neuron count, and decreased neurogenesis leading to cognitive dysfunction syndrome [91]. These changes, combined with mobility issues, often result in behavioral symptoms such as vacant stares and slowed movement [92].

Immune function deteriorates in dogs and cats with age [82], reducing vaccine efficacy [81] and increasing susceptibility to infections such as those caused by *Pasteurella* [6]. In the circulatory system, the incidence of heart disease is rises [69,83], and structural cardiac changes have been observed in older dogs, which may affect diastolic function [93]. Dermatological changes, including hair loss and dull coat, are also common [69]. These systemic alterations underscore the close link between health status and nutrition in aging pets. A thorough understanding of their physiological traits, coupled with an individualized nutritional approach, is therefore essential for promoting their wellbeing and quality of life.

### 4.2. Nutritional Requirements of Aging Dogs and Cats

The nutritional requirements of older companion animals differ significantly from those of younger adults owing to age-related physiological changes. Current leading nutritional guidelines, such as those issued outlined by the American Association of Feed Control Officials and the European Pet Food Industry Federation [3], are largely based on the 2006 National Research Council “Nutrient Requirements of Dogs and Cats” report [94]. However, research specifically addressing the needs of senior dogs and cats has advanced little in the past two decades. A review of the foundational studies cited in the 2006 report [94] reveals that subjects were primarily juvenile or adult dogs and cats under 6 years of age, with minimal focus on senior animals. Consequently, these authoritative guidelines may not be appropriate references for formulating diets tailored to older pets [3].

Establishing precise nutritional requirements for senior dogs and cats is complicated by factors such as age, breed, and physiological status. Nevertheless, recent studies on aging pets have revealed several consistent trends (Table 4). A key finding concerns water metabolism: total body water content declines with age [95], yet older animals often exhibit reduced renal sensitivity to arginine vasopressin (AVP), arginine vasopressin also called antidiuretic hormone (ADH), it help enhance renal water reabsorption [96]. Which means aging impairing thirst response and increasing the risk of dehydration [97]. Ensuring adequate water intake is therefore critical for senior pet health [98].

Protein requirements for senior dogs and cats remain contentious. Although some researchers argue that high-protein diets may burden the kidneys in human [99], others indicate that they can be safe or beneficial under certain conditions in cats [100]. For instance, one study found that an appropriately formulated high-protein diet (40% protein) did not exacerbate renal damage in senior cats and was associated with improved liver function and antioxidant metrics [9].

Despite the market trend toward high-protein pet foods (a crude protein content of 40% in commercial diets in China) [101,102], formulation should be guided by physiological need. Research providing 32% and 40% crude protein to senior dogs and cats, respectively, based on species-specific amino acid metabolism, underscores the importance of tailoring protein levels to individual metabolic profiles rather than following generalized trends [103].

Regarding fats, although digestion rates may not differ with age, senior dogs often show elevated blood lipid levels [85], suggesting that hyperlipidemia is a common concern in this population [104]. High-fat diets have been associated with accelerated aging [105], yet senior dogs are often deficient in ω-3 fatty acids, highlighting the need for supplementation with high-quality fats [104,106].

Carbohydrates are not essentials for dogs and cats, but both species can digest and utilize them efficiently [107]. Their strong gluconeogenic capacity allows conversion of protein to glucose, though at a metabolic cost, making digestible carbohydrates valuable for protein sparing [107,108]. Dietary fiber, often regarded as an anti-nutritional factor at high levels in livestock due to its tendency to reduce nutrient digestibility [109], has gained recognition for its health benefits in dogs and cats. Senior dogs and cats show improved apparent digestibility, including of calcium, with low to moderate fiber inclusion [85], supporting its judicious use in senior diets.

Additionally, knowledge of vitamin requirements in geriatric dogs and cats remains limited, though a consensus is emerging on the potential benefits of antioxidant supplementation (e.g., vitamins C and E) for healthy aging [110,111]. Research on mineral elements is scarcer. Phosphorus metabolism is of particular concern, as inorganic phosphates are increasingly added to enhance palatability [112]. A U.S. market analysis found 30% of canned and 10% of dry cat foods have a calcium-to-phosphorus ratio below 1.0 [113]. Elevated serum phosphate, driven by excessive inorganic phosphorus intake, is linked to chronic kidney disease (CKD) progression and increased cardiovascular risk [114,115,116]. The rising incidence of CKD in senior cats may therefore be related to the highly digestible inorganic phosphorus (e.g., H_3_PO_4_ and NaH_2_PO_4_) commonly used in commercial foods [117], however further research should be investigated to confirm this.

**Table 4:** Nutritional requirements of senior dogs and cats.

Nutrient	Changes in Demand (Compared to Adult Stage)	References
Water	Decreased demand, but prone to dehydration	[97]
Protein	A reasonable high-protein requirement	[9,103]
Fat	Total fat demand decreased, more omega-3 polyunsaturated fatty acids should be supplemented	[104]
Carbohydrates/Dietary Fiber	Carbohydrates remain necessary; appropriate dietary fiber should be provided	[85,107]
Vitamins	Increased demand of Vitamin C and Vitamin E	[110,111]
Minerals	Adequate calcium; reduced inorganic phosphorus intake	[114,115,116,117]

### 4.3. Current Nutraceuticals and Supplements for Aging Dogs and Cats

In companion animals, particularly dogs and cats, nutritional strategies to counteract aging often focus on enhancing specific dietary components, with antioxidants being a primary target. Dietary management through amino acid supplementation has also been explored for behavioral and metabolic regulation. For example, glycine supplementation has been shown help alleviate glutathione deficiency associated with oxidative stress in older cats [15]. A comparative study of younger (<3 years) and senior (>9 years) cats revealed significantly lower levels of total, oxidized, and reduced glutathione in the blood and red blood cells of older individuals. Supplementing senior cats with a diet containing 1.5% free glycine for 12 weeks increased baseline red blood cell glutathione levels and modified markers of oxidative stress [41].

Polyunsaturated fatty acids (PUFAs) have also received considerable attention as dietary supplements for aging pets. Studies indicate that n-3 PUFAs supplementation can improve the quality of fur, reduce inflammation [104], and mitigate cognitive decline and anxiety in senior animals [24,118,119]. A study of enriched diets and nutraceuticals for older dogs and cats noted that cognitive benefits from n-3 PUFAs are particularly evident at higher doses [119]. In one study, dietary inclusion of medium-chain triglycerides in aging Beagle dogs elevated PUFA levels in the parietal cortex, suggesting a potential mechanism for addressing age-related cognitive decline [120]. It is important to note that adequate vitamin E intake is essential when supplementing with PUFAs, as deficiency can reduce their efficacy [121,122].

In addition, the importance of dietary fiber for senior pets is growing, leading to increased demand for this nutrient. Studies indicate that incorporating ingredients like beet pulp, fructo-oligosaccharides, and galacto-oligosaccharides into the diets of older dogs improves fecal quality and promotes beneficial shifts in the gut microbiome [123]. Another study suggested that oligosaccharide supplementation can enhance fat digestibility and food intake, though it may slightly increase fecal moisture [124]. Pet food manufacturers appear to recognize this importance: a U.S. market survey found that foods for senior cats contained significantly higher crude fiber levels than those for adults, making it the only macronutrient that consistently differed between adult and senior formulations for both dogs and cats [125]. Furthermore, appropriate fiber inclusion can facilitate calcium absorption in senior pets, highlighting the need to consider interactions between macronutrients in diet formulation [85].

Beyond fiber, antioxidant vitamin supplementation not only supports health but can also extend the shelf-life of pet food and maintain product quality after opening [126]. Research on B vitamin supplementation exists, though studies often use mixed formulations, making it difficult to attribute benefits specifically to B vitamins [127,128]. The role of nutraceuticals in supporting joint health is also significant. Evidence suggests that undenatured type II collagen may be more effective than conventional glucosamine and chondroitin sulfate supplements in promoting joint health in companion animals, even at lower dosages [129].

Finally, feeding patterns themselves are gaining attention. Time-restricted feeding (TRF) has emerged as a promising dietary strategy. Empirical studies in dogs show that once-daily feeding, compared to more frequent meals, is associated with better cognitive function and a lower incidence of gastrointestinal, dental, orthopedic, and renal disorders [130]. These findings align with rodent studies linking TRF to improved metabolic health, circadian rhythm optimization, and increased longevity [130,131]. Together, this evidence underscores that when pets eat, alongside what they eat, plays a crucial role in supporting healthy aging and reducing the risk of age-related diseases.

The above indicates that extensive research has been conducted on the aging of dogs and cats. Regrettably, no nutritional guidelines for senior dogs and cats have been released to date; consequently, scholars often present fragmented findings in their discussions, which constitutes the second barrier to nutritional research on senior pets (Figure 1).

## 5. Research Projects About Aging Companion Animals in Recent Years

In recent years, several major research initiatives have been launched to study aging in companion animals. In the United States, the Dog Aging Project—led by researchers from institutions including Texas A&M University and the University of Washington—enrolled 976 pet dogs between January 2021 and July 2024 for a 50-week longitudinal study [132]. The project collects multi-omics data (DNA from cheek swabs, peripheral blood, urine, feces, and hair) alongside detailed health records, aiming to decipher aging mechanisms, identify biomarkers, and build open-access resources for the global research community [133]. While its decentralized sample collection poses challenges in standardization and diversity, the project has already generated substantial data advancing the study of behavior, nutrition, and healthcare in aging dogs, with multiple publications to date [134,135,136].

Another significant U.S. effort is the Golden Retriever Lifetime Study (GRLS), funded by organizations such as the Morris Family Foundation and the Blue Buffalo Cancer Research Foundation. Beginning in 2012, it enrolled 3044 Golden Retrievers aged 6 months to 2 years. As of May 2021, 2251 dogs remained active, with annual owner/veterinary questionnaires and biospecimen collection tracking lifestyle, environment, diet, and health outcomes [137]. The study has yielded insights into serum aging biomarkers, environmental drivers of tumorigenesis, and treatment patterns, with at least 16 peer-reviewed publications as of October 2025 [138,139].

In the United Kingdom, the Generation Pup project led by Dogs Trust is an ongoing lifelong cohort study open to puppies under 16 weeks. It examines how genetics, environment, diet, and social interactions influence health and behavior across the lifespan, with a long-term goal of tracking dogs into old age to identify risk factors for age-related conditions [140]. Other studies include the monitoring study on aging dogs conducted by NC State Veterinary Hospital [141], and the gut microbiota tracking study on 50 dogs and 145 cats conducted by Chinese Nourse Company [142], which were relatively smaller in scale and influence.

Alongside these large cohorts, smaller entities—including some small and medium-sized enterprises and research institutes—have conducted studies using dogs and cats from experimental facilities [9,24,127]. Although such settings allow more controlled sample collection and behavioral observation, they are often constrained by limited sample sizes and access to sufficient aged animals, posing challenges for developing and testing anti-aging nutritional interventions.

It follows that current mainstream research on canine and feline aging is survey-based cohort studies. These studies have large data volumes and no ethical risks but limit in-depth research, which is the third major barrier to senior pet research (Figure 1).

## 6. Future Directions in Research on Aging of Dogs and Cats

### 6.1. Develop Combined Biomarkers Based on Non-Invasive Detection Technology

The strong emotional bonds between humans and their companion animals elevate ethical considerations, often prioritizing animal welfare over invasive procedures more commonly accepted in traditional laboratory or farm animal studies. This ethical priority presents a significant challenge: balancing the need to prevent harm with the pursuit of robust scientific data.

Consequently, a substantial portion of companion animal research relies on observational markers or minimally invasive sampling, such as blood, hair, and feces. While crucial for welfare, this limits the use of more traditional, invasive techniques like tissue biopsies, which can provide deeper mechanistic insights. To bridge this gap, the development and adoption of sophisticated non-invasive health monitoring technologies is paramount.

Recent advancements in this area are promising. In physiological monitoring, devices like the 3D ultrasound Bladder Scan offer a non-invasive alternative to catheterization for assessing hydration status [143]. Similarly, flash glucose monitors enable real-time metabolic tracking in diabetic pets, despite current challenges with sensor reliability [144]. Tools like non-invasive intracranial pressure monitors, though designed for neurology, hold potential for studying nutritional impacts on brain health [145].

For behavioral and metabolic correlation, wearable technologies such as the PetPace smart collar can objectively track activity and sleep patterns, indirectly reflecting energy balance [146]. Future progress lies in integrating these multimodal data streams—combining metabolic, physiological, and behavioral metrics—to overcome current limitations in device accuracy and single-indicator focus.

However, a critical bottleneck remains. While these technologies excel at collecting real-time data, there is an urgent need to develop corresponding aging biomarkers that are compatible with non-invasive data sources, such as behavior patterns, fecal analysis, or data from implantable chips. The integration of AI with these novels, ethically aligned monitoring frameworks is therefore set to define the future direction of nutrition research for aging dogs and cats.

### 6.2. Focus on the Common Nutritional Needs and Personalized Nutrition of Aging Dogs and Cats

The nutritional requirements of aging companion animals exhibit substantial individual variation. This diversity stems from age-related shifts in nutrient metabolism, combined with factors such as breed, size, and underlying health status [2]. For example, the nutritional needs of large-breed dogs often differ from those of smaller breeds in their senior years [147,148], and predisposition to specific age-related diseases further individualizes dietary demands [149].

In practice, maintaining optimal health in aging dogs and cats is challenging. Innovations in personalized nutrition aim to address this by tailoring dietary interventions to the individual—considering genetics, health metrics, and lifestyle—to better support healthy aging and prevent frailty [150,151]. The integration of artificial intelligence with telemedicine also holds promise, enabling remote monitoring and personalized dietary management for pets with chronic conditions [152]. However, the current development of precision nutrition is hampered by a lack of foundational data. A comprehensive understanding of breed-specific needs and aging physiology is essential, yet remains a significant research gap.

Given the recent launch of multiple large-scale aging studies in dogs and cats [132,137,140]—alongside smaller laboratory-based research—there is now an opportunity to systematically integrate these findings. Building on this foundation, the development of evidence-based nutritional guidelines for healthy aging would represent a crucial advance in the field [3]. The key priority for future research should be to accurately obtain data on dogs and cats of different ages and breeds from cohort studies, and to use meta-analysis to analyze their nutritional requirements across different breeds and physiological states (age, sex, and health status), as well as the effects of various anti-aging interventions applied to them. Such an effort would help unify currently fragmented research and provide a clear framework for future studies aimed at optimizing the health and longevity of aging companion animals.

### 6.3. Conduct Cross-National and Cross-Regional Research on Elderly Dogs and Cats

Whether conducting large-scale cohort studies or evaluating the efficacy of nutritional supplements for aging pets, experimental verification remains an indispensable stage. This requires maintaining a sufficient population of elderly dogs and cats for scientific purposes. However, given the practical and ethical complexities of caring for senior animals, individual research institutions often have limited capacity to house such cohorts. This reality underscores the necessity of collaborative research across institutions and international borders.

Many researchers are positively inclined toward such collaborative models. Nevertheless, practical implementation faces considerable challenges, including logistical risks in cross-border sample transfer, variability in breeding environments, and differing animal welfare standards. To advance the field, it is imperative for all stakeholders—researchers, institutions, and policymakers—to work together in reducing policy and infrastructural barriers. Only through coordinated effort can robust multinational and cross-regional research on aging companion animals be effectively realized.

## 7. Conclusions

This review synthesizes current knowledge on the nutrition of aging companion animals, identifying key advances and critical gaps in the field. Although extended lifespans in dogs and cats have increased the focus on senior pet health, research into their nutritional requirements remains underdeveloped, constrained by the absence of reliable aging biomarkers, fragmented study designs, and a scarcity of healthy aged animal models. We consolidate evidence across multiple biological levels—from molecular and cellular to systemic and microbial—to outline current biomarkers of aging and integrate findings on physiological changes, nutrient needs, and commonly used dietary supplements in senior pets. The review also examines recent large-scale cohort studies dedicated to aging in these species. Moving forward, we propose three strategic priorities to advance the field (See Figure 1): (1) developing aging biomarkers through non-invasive methods, (2) establishing evidence-based nutritional guidelines to support healthy aging, and (3) fostering cross-border collaboration to enable personalized nutrition research. Implementing these directions will help build a stronger scientific foundation for improving the healthspan and quality of life of aging dogs and cats.

## Figures and Tables

**Table 1:** Age Classification of senior dogs and cats by major organizations and institutions.

Classification	Age (Years)	Reference
Cat	>10	AAHA [11]
	>11	iCatCare [12]
	>12	Case et al. [13]
	>12	Salt et al. [14]
Small breed dog	>10	FEDIAF [15]
	>11.5	Case et al. [13]
	>12	Salt et al. [14]
Medium breed dog	>10	Case et al. [13]
	>10	Salt et al. [14]
Big breed dog	5–8	FEDIAF [15]
	>7.5	Case et al. [13]
	>10	Salt et al. [14]

AAHA, American Animal Hospital Association; FEDIAF, European Pet Food Industry Federation; iCatCare, International Cat Care.

**Table 2:** Biomarkers for aging research in dogs and cats.

Type	Biomarkers	Animal	References
Molecule	DNA damage	dog	[19,20]
		cat	[19]
Molecule	Telomere length	dog	[21,22,23]
Molecule	Aβ1-42, and/or Aβ42/40 ratio, AβpN3	dog	[24,25,26,27]
Molecule	neuronal cytoskeletal protein neurofilament light chain (NfL)	dog	[28,29]
Molecule	P16	cat	[30]
		dog	[31]
Molecule	DNA methylation	cat	[32]
		dog	[33,34,35]
Molecule	osteoprotegerin	dog	[36,37]
Molecule	angiotensin II	dog	[36,37]
Molecule	endothelin-1	dog	[36,37]
Molecule	osteocalcin	dog	[38,39]
Molecule	carboxyterminal propeptide of type-I procollagen	dog	[38,39]
Molecule	malondialdehyde	dog	[20,40]
Molecule	glutathione	cat	[41,42]
Molecule	8-hydroxy-2-deoxyguanosine	dog	[42,43]
Molecule	P21	dog	[44,45]
Molecule	glucose metabolism-related: adiponectin, free fatty acids/FFA ^1^	dog	[46]
Molecule	Liver function-related: alkaline phosphatase/ALP, alanine aminotransferase/ALT ^1^	dog	[46]
Cell	CD8^+^ T cells	dog	[31,47,48,49]
Cell	CD4^+^ T cells	dog	[47,48,49]
Cell	CD4:CD8 ratio	dog	[47,48,49,50]
Organ	Global glomerulosclerosis, interstitial fibrosis, and tubular atrophy	dog	[51]
Organ	brain amyloid accumulation	dog	[52]
Organ	Age-related cataract	dog	[53]
Gut Microbiota	*Faecalibacterium*	dog	[54,55]

^1^ The literature lists 12 molecules significantly associated with aging, while this table includes only those with a correlation coefficient greater than 0.30.

**Table 3:** Changes in physiological characteristics of senior dogs and cats.

System	Physiological Changes
Overall	Increased tumor incidence [68]
Digestive System	Increased tooth loss/oral diseases [15,75] Diminished taste/smell [15] Decreased digestive capacity [69] Altered gut microbiota structure [15,76,77]
Musculoskeletal	Causing osteoarthritis [78], joint degeneration [69], chronic pain [69]
Nervous System	Decline in cognitive abilities [79,80] Brain atrophy, neuron loss, decreased neurogenesis [80]
Immune System	Decreased vaccine efficacy [81] Reduced immune capacity [82]
Circulatory System	Increased heart disease [69,83]
Skin and Coat	Hair loss, thinning of the coat, and a loss of luster [69]

## Data Availability

Not applicable, this is a review based on the references from PubMed.
